# Research on the characteristics and protection of water hammer in long-distance dual-pipe water supply systems

**DOI:** 10.1371/journal.pone.0314998

**Published:** 2024-12-17

**Authors:** Xiaolei Zhang, Xiaoyi Guo, Yading Chen, Chen Yang, Shuyu Liu, Lixia Guo

**Affiliations:** 1 School of Water Conservancy, North China University of Water Resources and Electric Power, Zhengzhou, Henan, China; 2 Henan Provincial Key Laboratory of Hydrosphere and Watershed Water Security, Zhengzhou, Henan, China; 3 Zhangjiagang Zhangshui Project Management Co., Ltd, Suzhou, Jiangsu, China; 4 Collaborative Innovation Center for Efficient Utilization of Water Resources, Zhengzhou, Henan, China; 5 Hohai University, College of Water Conservancy and Hydropower Engineering, Nanjing, Jiangsu, China; Izmir Katip Celebi University: Izmir Katip Celebi Universitesi, TÜRKIYE

## Abstract

Hydraulic transients in long-distance pressurized water pipelines significantly impact their normal operation. This study develops a one-dimensional mathematical model for pressurized water pipelines using the method of characteristics and incorporates water hammer equations for dual-pipeline systems. The model is validated with experimental data, and simulations are conducted under real engineering conditions, focusing on valve closure operations. The analysis examines the transient responses for varying valve closure times (*T*) and the effect of installing surge tanks. Results show that increasing valve closure time and installing surge tanks both mitigate water hammer impacts. Specifically, when valve closure time exceeds 300 seconds, surge tanks reduce maximum pressure below the pipeline’s tolerance (*Pmax*) and decrease the number of nodes experiencing damaging negative pressures. This model effectively simulates hydraulic transients in dual-pipeline systems and provides a foundation for developing protective measures for pipeline operations.

## 1 Introduction

During the pressurized water conveyance in long-distance pipelines, the changes in liquid flow within the pipeline have a significant impact. Rapid changes in the flow state within the pipeline are detrimental and can lead to destructive consequences for the pipeline [1,2]. The transition between operational conditions, the starting and stopping of water pumps, and the opening and closing of valves lead to hydraulic transients in the pipeline. When a water pump is suddenly shut down, it results in pump stoppage water hammer, which can cause negative pressure in the pipeline and lead to transient cavitation flow [3], ultimately resulting in pipeline rupture [4]; When a valve is abruptly closed, it results in valve closure water hammer. This phenomenon causes an increase in pipeline pressure, and if the pressure exceeds the pipeline’s pressure-bearing capacity, it can lead to pipe bursting. This is one of the most dangerous hydraulic phenomena in pressurized pipeline systems [5]. Now long-distance water transmission in order to improve the water supply security of water pipelines, usually choose to lay two water pipelines side by side and set up a connecting pipe connection, double-pipe section of water pipeline changes in hydraulic conditions are more complex, for long-distance double-pipe section of the hydraulic conditions within the transient changes in the phenomenon of the study is more urgent.

The methods to mitigate the maximum pressure generated during valve closure water hammer in pipelines mainly focus on the following aspects: air chamber, surge tanks, air valves, bypass pipes, check valves, and other pipeline measures [6]. Researchers have conducted extensive studies on the protective effects of these measures. Air valves are essential pressure regulating devices in pipeline water supply projects. They are not only used for automatic air intake and exhaust during pipeline filling and venting processes to prevent water hammer damage, but also for automatic air intake and exhaust during power failure and hydraulic transient processes in pump stations to prevent liquid vaporization and liquid column coalescence water hammer damage [7]. The air chamber is a mechanical device in a pumping pipeline that can reduce positive water hammer pressure and increase negative water hammer pressure [8], Yazdi et al. [2] found that through optimization of the position and size of air valves and air chambers: the air chamber is an effective measure for protecting against water hammer in pipelines, while the air valve has a relatively poor effect on preventing water hammer in pipelines. Kim et al. [1] explored the effect of proper selection of air chamber size and orifice inner diameter on reducing water hammer through field measurements and numerical simulations The effect of proper selection of air chamber size and orifice inner diameter on water hammer was investigated. However, air chambers are commonly used in situations where equipment flow is small, head is high, and there is a wide range of pressure control [9], But are characterized by high cost and maintenance expenses. Regarding the installation of bypass pipes as an engineering measure, installing bypass pipes in the pipeline can generate a certain amount of discharged water when water hammer occurs and water flows backward [10]. If the discharged water volume is too large, it not only causes economic waste but also affects the safety of pump stations and surrounding buildings [11]. Check valves play a role in blocking the backflow of water inside the pipeline after water hammer occurs. However, they do not provide protection against the damage caused by excessively high positive or negative pressures within the pipeline [12]. The design of surge tanks aims to mitigate the impact of water hammer on pressure pipelines by regulating the fluctuation of water levels inside the well, thereby protecting the upstream section from the effects of pressure surges. Another function is to replenish the pipeline when a vacuum occurs in the pipe, creating negative pressure [13].

For large-scale long-distance water pipeline projects, numerical simulations are required during construction to assess the safety of water conveyance in the pipeline. If water hammer protection devices are to be installed, it is necessary to determine the dimensions and positions of these protective devices. Simulations are then conducted to ensure the effectiveness of the protective measures against water hammer in the pipeline. Therefore, when installing surge tanks in the pipeline, it is necessary to consider the shape, size, and location of the surge tanks. Firstly, surge tanks in pipelines are primarily used to reflect pressure waves or to store water resources to reduce the magnitude of pressure fluctuations within the pipeline [12,14]. In the simulation of surge tanks, Kendir et al. [15] and others used numerical simulation methods to model the types of surge tanks in hydroelectric power plants and determined the optimal surge tank type. Sarasúa et al. [16] and others established a dynamic model for a water and hydroelectric power station with pressurized pipelines and surge tanks. Through stability analysis, they determined the design area for surge tanks. Chen et al.[17] optimized the placement of surge tanks in pipelines. They focused on the impact of the impedance hole size on water hammer and provided a reasonable range for impedance hole values. The theoretical analysis results were validated through engineering examples. Guo et al. [18] suggested that the initial height inside the surge tank affects its pressure damping effect and conducted simulations. Cheng et al. [19] calculated the impact of surge tank connectivity pipe length on water hammer protection using the method of characteristics. The above-mentioned studies mostly analyze the impact of specific characteristics of surge tanks on their effectiveness. There is relatively less research on the effectiveness of surge tanks under different operating conditions.

Although many people have done a lot of research on water hammer protection in pipelines, few scholars have conducted water hammer protection research on the working condition of water transmission in double pipe sections. This study focuses on the protection against water hammer in pipeline segments under special conditions, specifically in the context of a common configuration of long-distance water transmission involving dual pipeline segments. The extreme condition considered is the closure of the terminal valve. The methods employed for water hammer protection include extending the valve closure time (T) and adding surge tanks within the pipeline segments. By adjusting the valve closure time and strategically installing surge tanks, the goal is to reduce the positive pressure generated by water hammer resulting from the closure of the terminal valve. The analysis focuses on studying the pressure changes at nodes in another pipeline segment following the occurrence of water hammer due to the closure of the terminal valve during operational conditions. This aims to assess the effects of prolonging valve closure time and installing surge tanks.

## 2 Mathematical model and validation

### 2.1 dual-pipeline water conveyance

**Fig 1:**
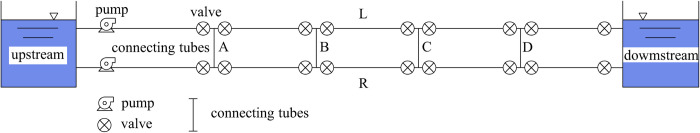
Schematic diagram of dual-pipeline water conveyance.

In this study, dual-pipeline water conveyance refers to an engineering project where water is simultaneously pumped from a single upstream reservoir to a single downstream reservoir. To ensure the water transfer from the upstream to the downstream reservoir during maintenance, a bypass pipe is opened between the two pipelines. This allows the water to be rerouted, avoiding the section under maintenance and ensuring normal water conveyance. (**Fig 1)** illustrates the installation setup of the bypass pipe in the dual-pipeline water conveyance system studied (In reality, the two pipelines are arranged side by side at the same elevation.). Under normal operating conditions, the upstream reservoir through the two pumps to the downstream water transfer, parallel to the two pipe segments are responsible for water transfer tasks, the two pipe segments set up between the four connecting pipe, in the connecting pipe and the main section of the two ends are set up with valves, responsible for closing or opening the connecting pipe.

### 2.2 Method of characteristics

The fundamental differential equations for water hammer comprise the motion equation and continuity equation. Due to the pipe’s excessive length and minimal elevation changes, the segment where water hammer occurs is treated as a horizontal pipe. Additionally, as the variations in flow rate and head with respect to position are much smaller than those with respect to time, the corresponding terms in both equations are neglected. After simplification, the equations are represented as (1) and (2).

$$\frac{\partial Q}{\partial t}+gA\frac{\partial H}{\partial x}+\frac{fQ|Q|}{2DA}=0$$ (1)

$$gA\frac{\partial H}{\partial t}+a^{2}\frac{\partial Q}{\partial x}=0$$ (2)

Where *Q* is flow rate(m^3^/s); *D* is pipe diameter; *A* is pipe cross-sectional area; *f* is pipe wall friction coefficient; *a* is water hammer wave speed; H is total water head; *x* is water hammer wave propagation distance; *t* is time; *g* is gravitational acceleration.

Let:

L=(1)+λ(2) (3)

Where *λ* is an assumed real number.

Substitute Eq (3) into Eqs (1) and (2):

$$L=\frac{\partial Q}{\partial t}+\lambda a^{2}\frac{\partial Q}{\partial x}+\lambda gA\frac{\partial H}{\lambda \partial x}+\frac{\partial H}{\partial t}+\frac{fQ|Q|}{2DA}=0$$ (4)

By full differentiation:

$$\{\begin{array}{c} \frac{dQ}{dt}=\frac{\alpha Q}{\alpha t}+\frac{\alpha Q}{\alpha x}\frac{dx}{dt}\\ \frac{dH}{dt}=\frac{\alpha H}{\alpha t}+\frac{\alpha H}{\alpha x}\frac{dx}{dt} \end{array}$$ (5)

When:

$$\lambda a^{2}=\frac{dx}{dt}=\frac{1}{\lambda }$$ (6)

Then:

$$L=dQ+\lambda gAdH+\frac{fQ|Q|}{2DA}dt=0$$ (7)

Solving Eq (6) yields two eigenvalues:

$$\frac{dx}{dt}=\pm a$$ (8)

**Fig 2:**
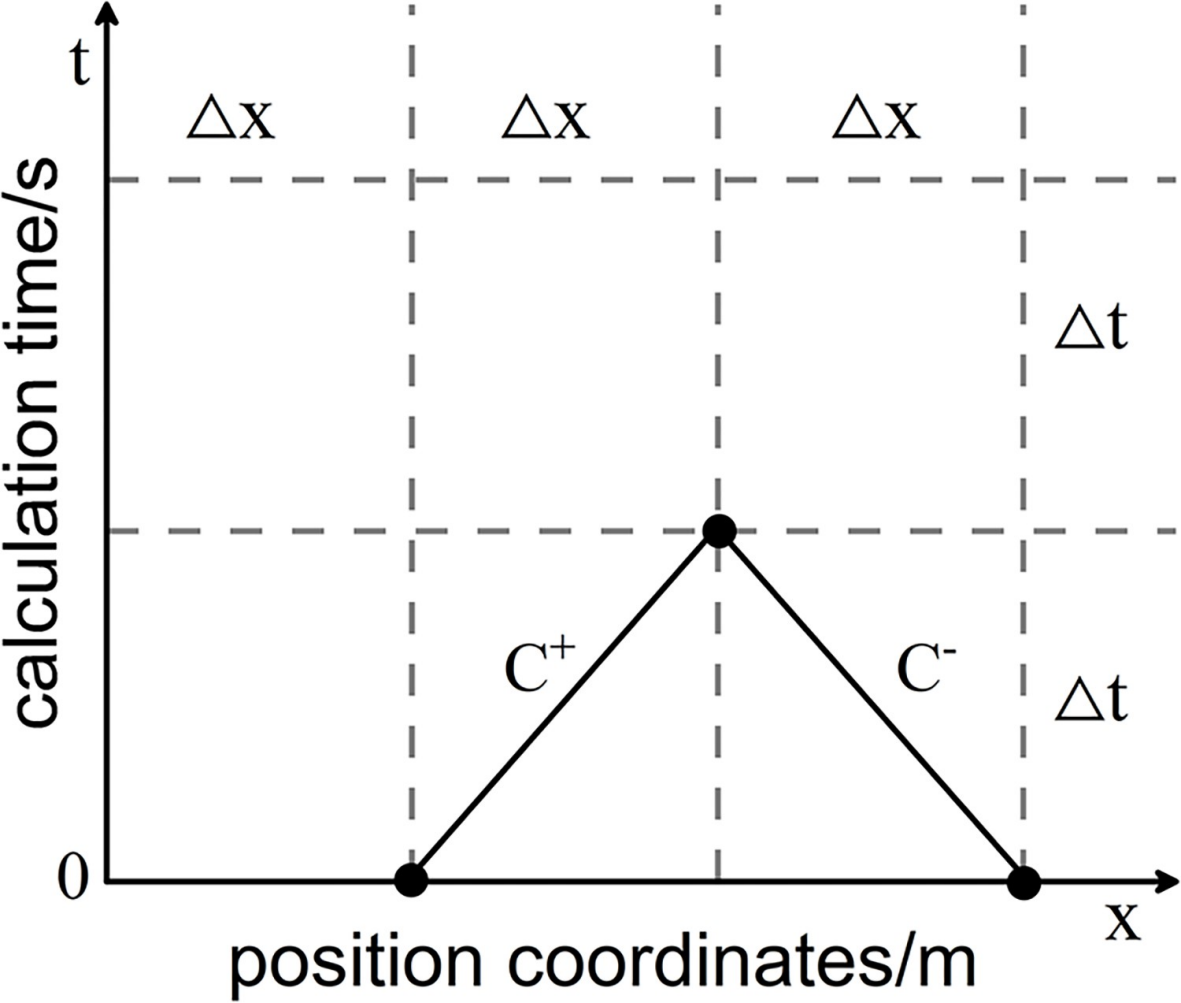
Schematic diagram of characteristic lines and grid division.

According to the schematic diagram of the characteristic line method shown in (**Fig 2**). The characteristic line Eqs 9 and 10 can be obtained from the results obtained from Eq 8, equation is as follows:

in the C+ direction:

$$\{\begin{array}{c} L=\frac{dQ}{dt}+\frac{gA}{a}\frac{dH}{dt}+\frac{fQ|Q|}{2DA}=0\\ +a=\frac{dx}{dt} \end{array}$$ (9)

in the C- direction:

$$\{\begin{array}{c} L=\frac{dQ}{dt}+\frac{gA}{a}\frac{dH}{dt}+\frac{fQ|Q|}{2DA}=0\\ -a=\frac{dx}{dt} \end{array}$$ (10)

C^+^:

$$H_{P}=C_{A}-S_{A}Q_{P}$$ (11)

C^−^:

$$H_{P}=C_{B}-S_{B}Q_{P}$$ (12)

Where H_P_ is the head of pressure pipe at point P; C_A_, S_A_ are the parameters of the C^+^feature line; C_B_, S_B_ are the parameters of the C^-^ feature line.

Solving the characteristic line equation using finite difference method, the results are as follows:

$$Q_{P}=\frac{C_{A}-C_{B}}{S_{A}+S_{B}}$$ (13)

Based on the basic hydraulic parameters *Q*_*A*_, *H*_*A*_, *H*_*B*_, △*t* at time t_0_ for points A and B, and utilizing Eq (11), one can solve for the *Q*_*P*_, *H*_*P*_ at point P at time *t*_*0*_+△*t*. Similarly, the *Q*_*P*_, *H*_*p*_ for all nodes can be determined.

### 2.3 Characteristics method for solving the two-pipe water transmission model

**Fig 3:**
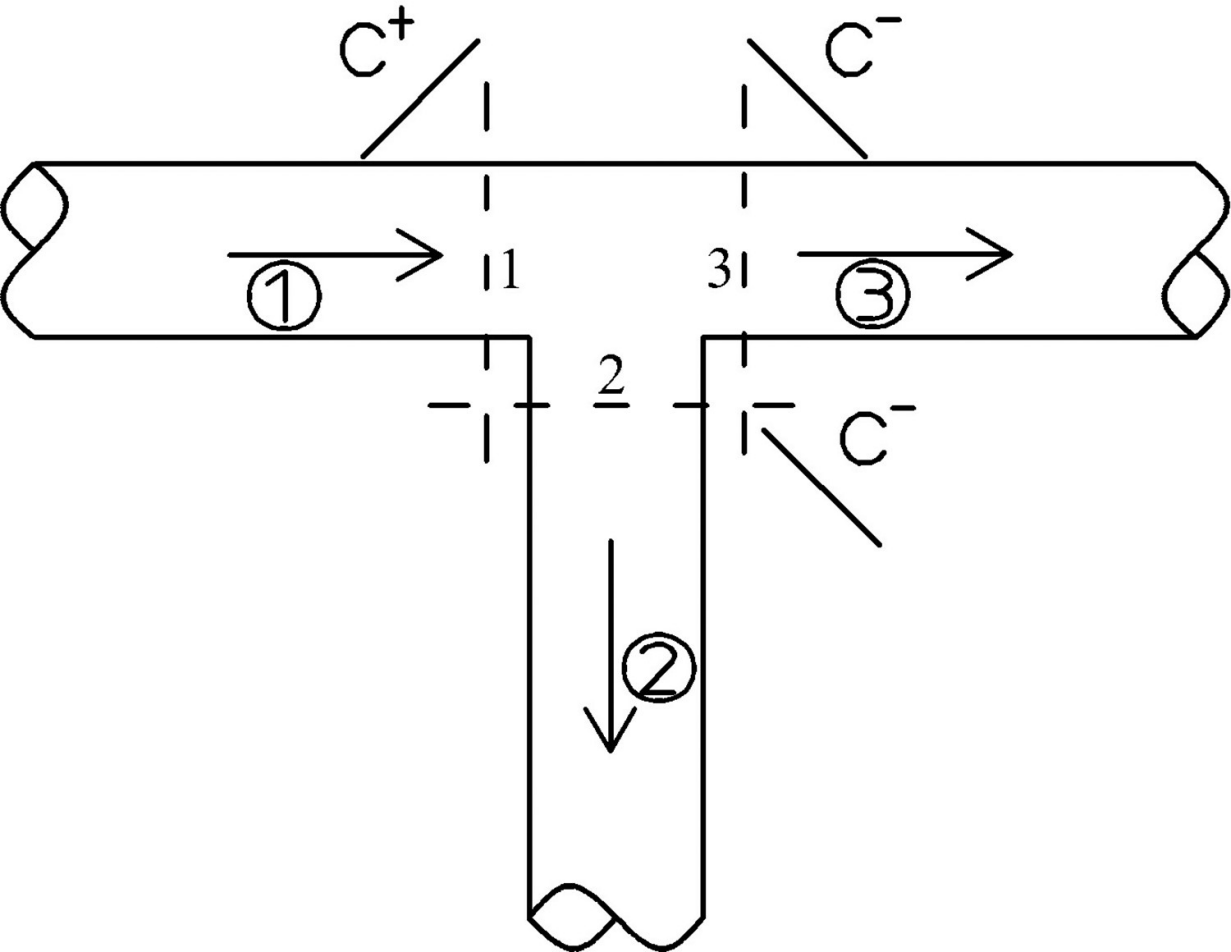
Condition 1 of the connecting tube.

Boundary condition at the connection pipe 1:

When the connecting pipe is as shown in the pipe section of **(Fig 3)**, the continuity and hydrologic conditions satisfied at the connection point, respectively, are shown below:

Continuity conditions

$$Q_{1}=Q_{2}+Q_{3}$$ (14)

hydrologic conditions

$$H_{P}=H_{3}=H_{2}=H_{1}$$ (15)

C^+^:

$$H_{P}=C_{P1}-S_{1}Q_{1}$$ (16)

Then:

$$Q_{1}=\frac{C_{P1}-H_{P}}{S_{1}}$$ (17)

C^−^:

$$H_{P}=C_{M3}+S_{3}Q_{3}$$ (18)

Then:

$$Q_{2}=\frac{H_{P}-C_{M2}}{S_{2}}$$ (19)

$$Q_{3}=\frac{H_{P}-C_{M3}}{S_{3}}$$ (20)

Bringing Eqs 15, 17, 18 into Eq 12:

$$H_{P}=\frac{\left(\frac{C_{M3}}{S_{3}}-\frac{C_{M2}}{S_{2}}+\frac{C_{P1}}{S_{1}}\right)}{\left(\frac{1}{S_{3}}-\frac{1}{S_{2}}+\frac{1}{S_{1}}\right)}$$ (21)

**Fig 4:**
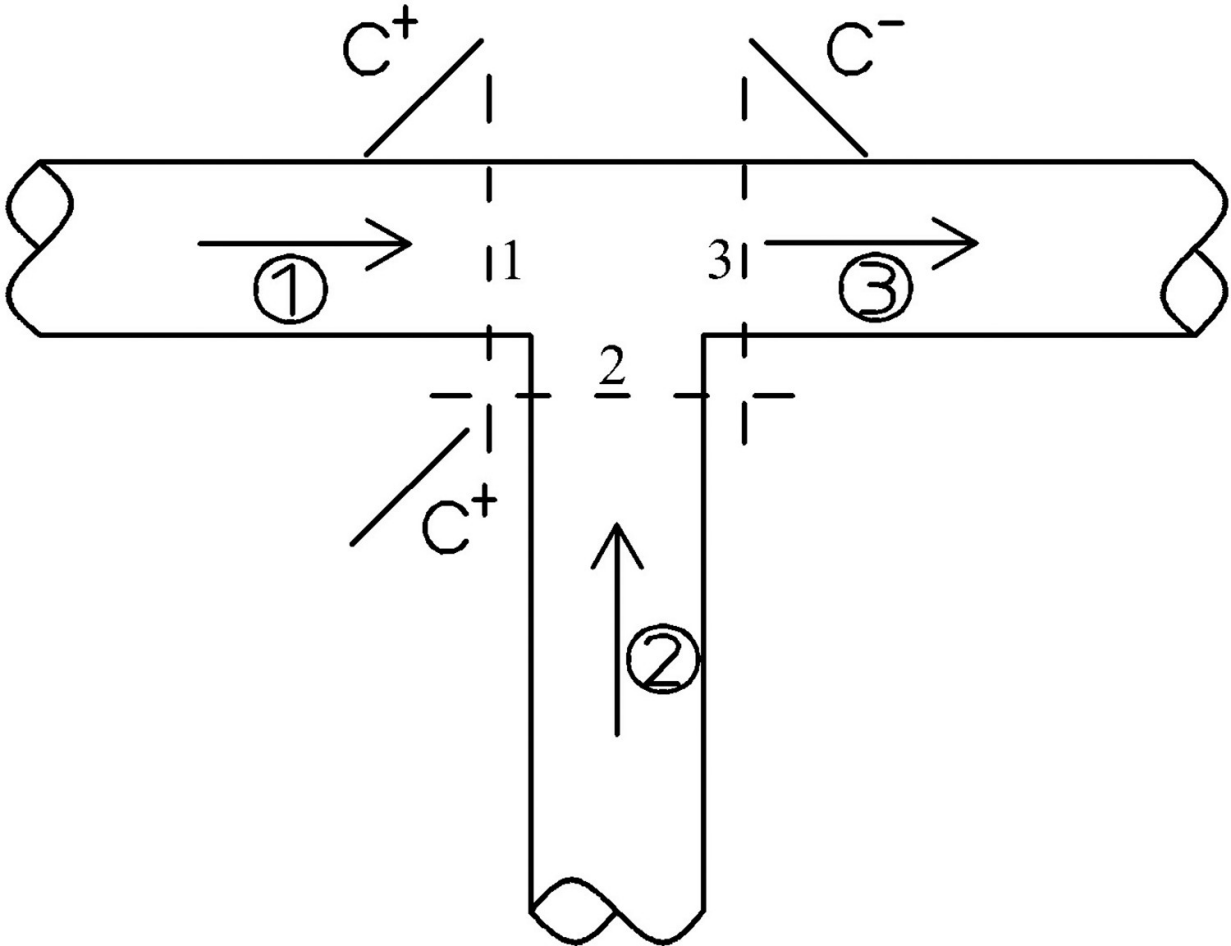
Condition 2 of the connecting tube.

When the connecting pipe is as shown in the pipe section of **(Fig 4)**, the continuity and hydrologic conditions satisfied at the connection point, respectively, are shown below:

Continuity conditions

$$Q_{3}=Q_{1}+Q_{2}$$ (22)

hydrologic conditions

$$H_{P}=H_{3}=H_{2}=H_{1}$$ (23)

C^+^:

$$H_{P}=C_{P1}-S_{1}Q_{1}$$ (24)

$$H_{P}=C_{P2}-S_{2}Q_{2}$$ (25)

Then:

$$Q_{1}=\frac{C_{P1}-H_{P}}{S_{1}}$$ (26)

$$Q_{2}=\frac{C_{P2}-H_{P}}{S_{2}}$$ (27)

C^−^:

$$H_{P}=C_{M3}+S_{3}Q_{3}$$ (28)

Then:

$$Q_{3}=\frac{H_{P}-C_{M3}}{S_{3}}$$ (29)

Bringing Eqs 24, 25, 27 into Eq 20:

$$H_{P}=\frac{\left(\frac{C_{M3}}{S_{3}}+\frac{C_{P2}}{S_{2}}+\frac{C_{P1}}{S_{1}}\right)}{\left(\frac{1}{S_{3}}+\frac{1}{S_{2}}+\frac{1}{S_{1}}\right)}$$ (30)

### 2.4 Boundary conditions of the surge tank

in the C+ direction:

$$H_{P1}=C_{P1}-S_{1}Q_{P1}$$ (31)

in the C- direction:

$$H_{P2}=C_{M2}-S_{2}Q_{P2}$$ (32)

$$H_{P1}=H_{P2}=H_{PS}$$ (33)

$$Q_{P1}-Q_{P2}=Q_{PS}$$ (34)

$$\frac{dZ}{dt}=\frac{1}{F}Q_{S}$$ (35)

$$Z=H_{PS}-K{Q_{S}}^{2}$$ (36)

Where subscripts 1 and 2 respectively denote the parameters of the pipeline before and after installing the surge tank; Qs is inflow to the surge tank; Z is water level in the surge tank; K is resistance coefficient of water flow entering the surge tank.

**Fig 5:**
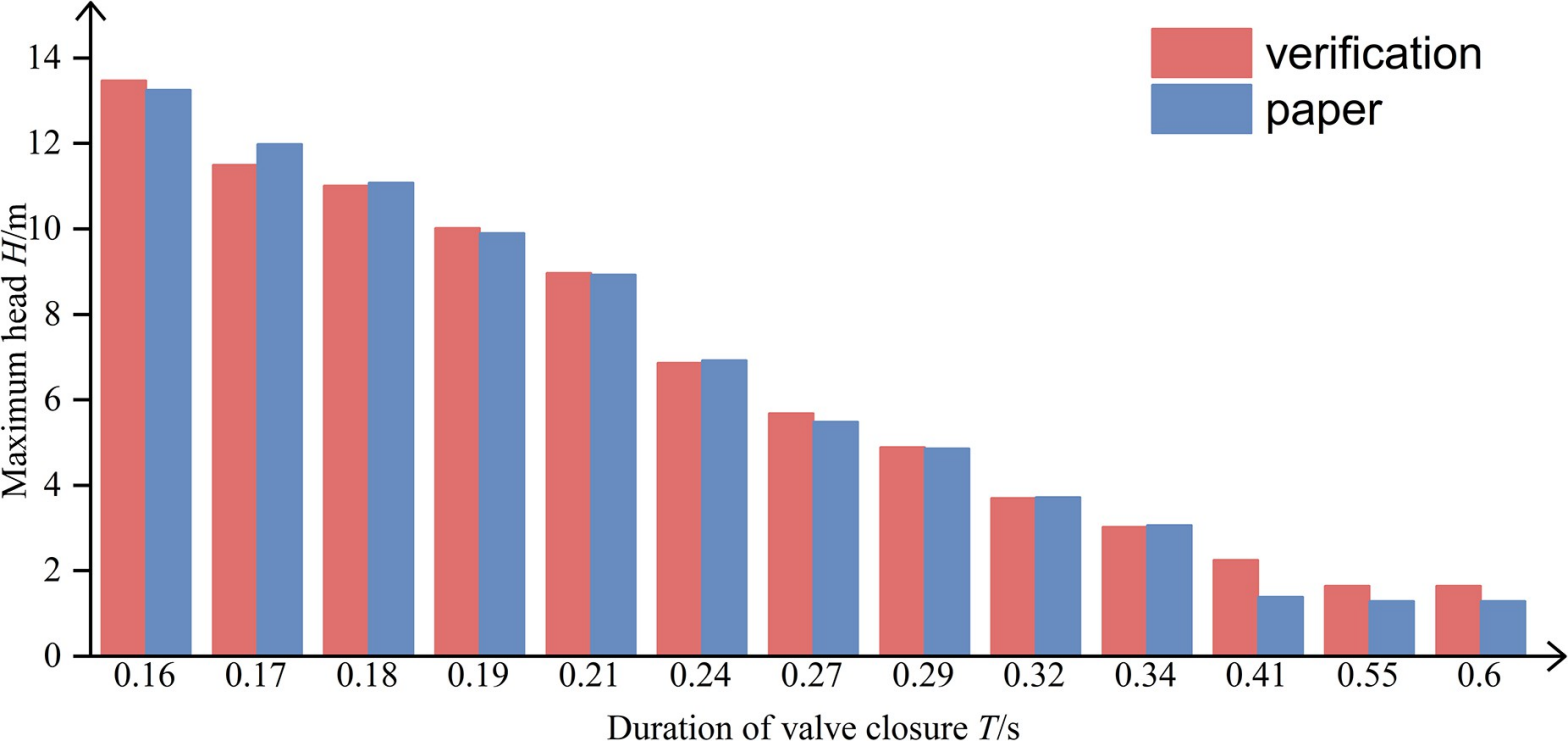
Verification results.

In this study, the measurement experiments of water strikes are used to validate the measurement experiments of water strikes published by Yi Sha in the Journal of Experimental Mechanics, "Measurement of water strike pressure waves in circular pipe flow and hydraulic calculations" [20]. Firstly, its physical experimental model is mathematically modeled, and the same working condition as the reference experiment is set, i.e., the maximum head value in the pipe section is derived under different valve closing times. The mathematical model is validated by the results of the two models, and the validation results are shown in **(Fig 5)**, which show that the model results are well validated with the results of the thesis under different valve shut-off times, and the Nash’s efficiency coefficient is 0.99, which indicates that this model can be used to simulate the shut-off valve water hammer, and the modeling parameters are set reasonably.

## 3 Mathematical modeling and operational condition setup

**Fig 6:**
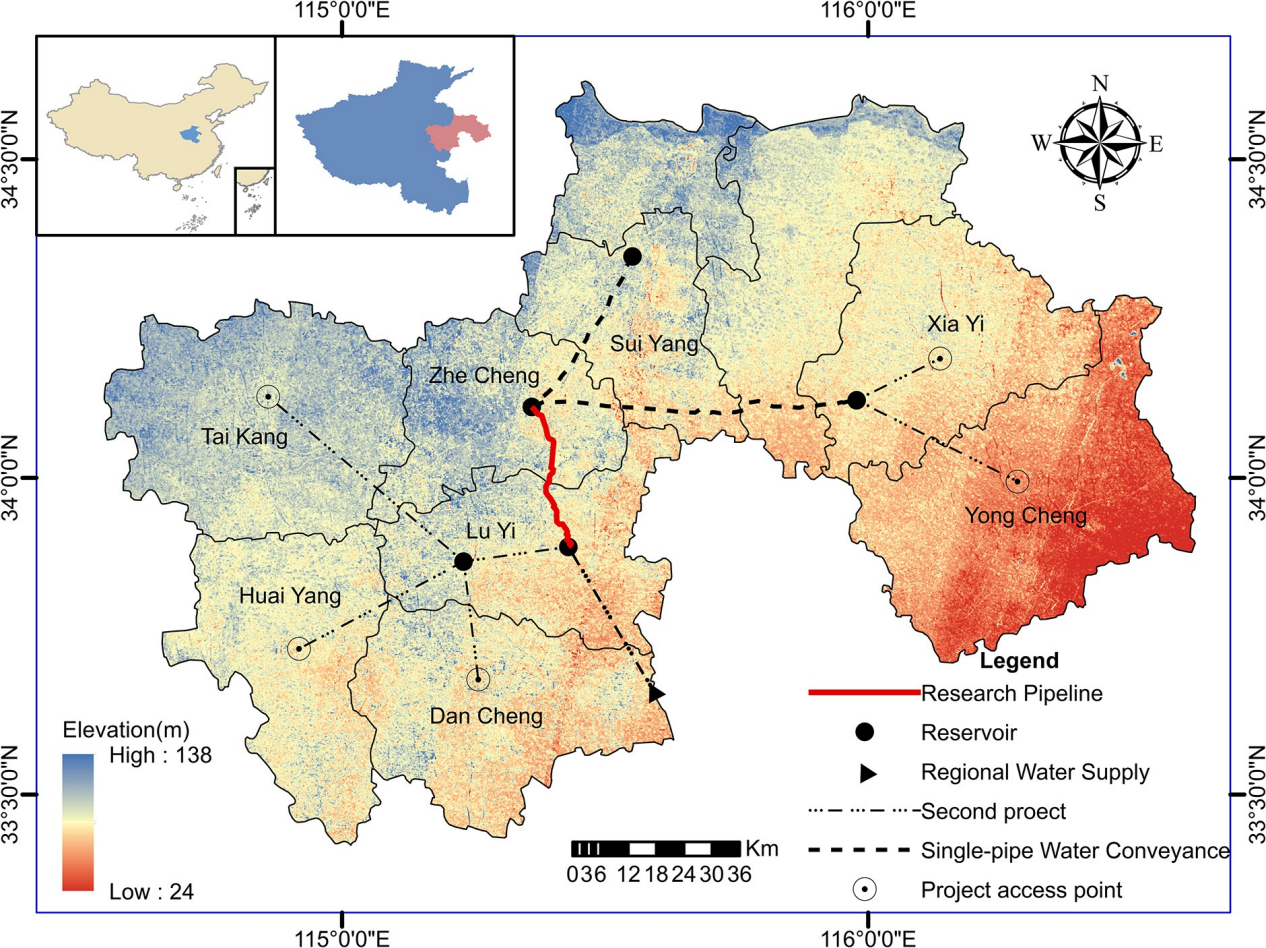
Engineering location map. Created by ArcGIS 10.6 software (https://www.arcgis.com).

**Fig 7:**
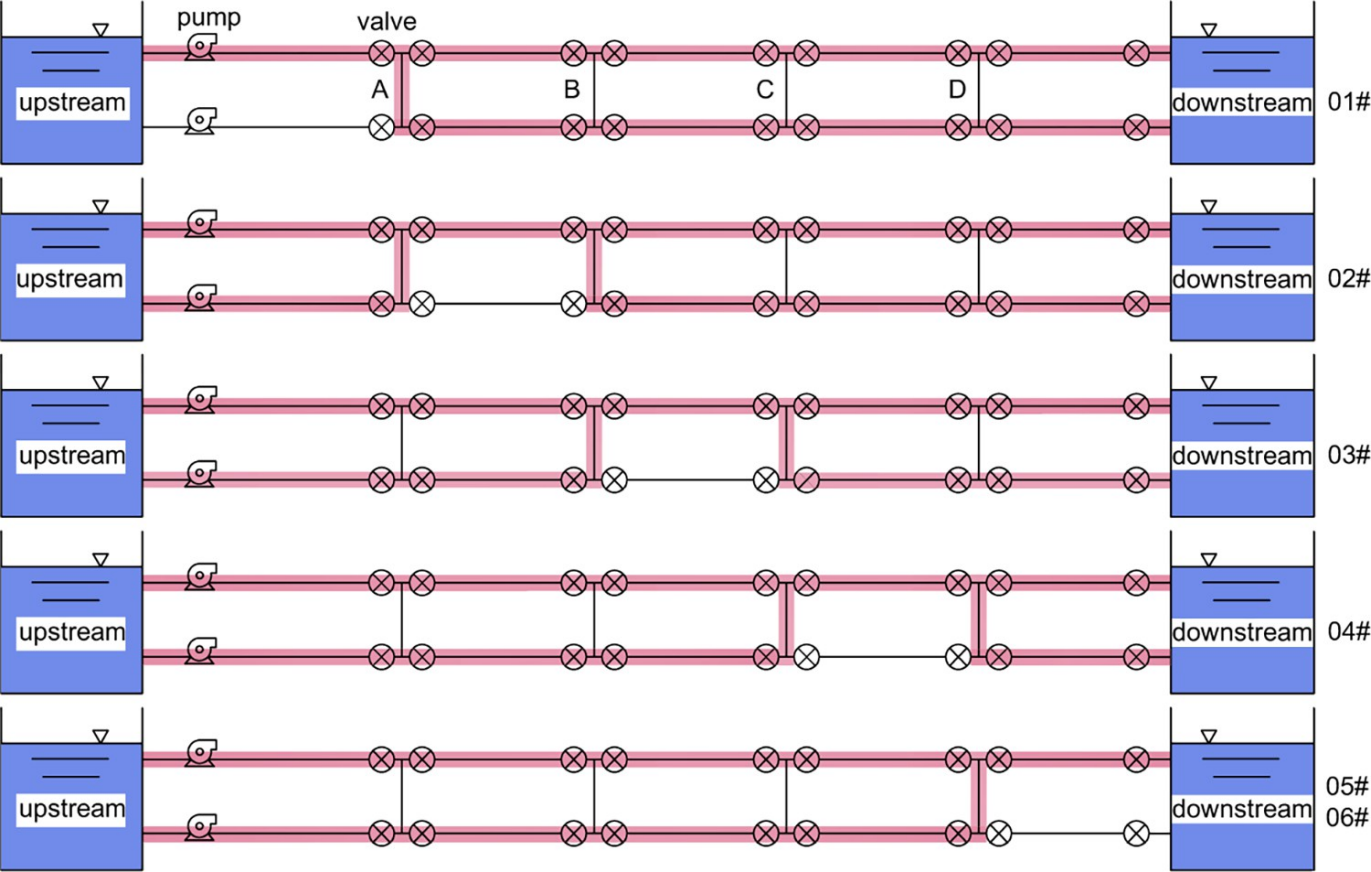
Schematic diagram of repair for different damaged sections in the dual pipeline water conveyance system.

The pipeline segment studied in this research is a section of the domestic long-distance water transmission project, the "Yinjiang-Jihuai Project." The project location is depicted in **(Fig 6)**, The pipeline delivers water from the Houchenlou Storage Reservoir in Luyi upstream to the Qiliqiao Storage Reservoir in Zhecheng downstream. representing a pressurized dual-pipe water transmission system. The pipeline, connecting an upstream reservoir to a downstream reservoir, transports water through pump stations. The configuration of the dual-pipe segment is illustrated in **(Fig 7),** where the left pipe segment, numbered as L from upstream to downstream, is connected to the right pipe segment, numbered as R. Furthermore, a four-section connecting pipe (designated as A, B, C, D) is employed to link the dual-pipe segments for water transmission. The dual-pipe segment is divided into five sections. In the event of any damage requiring maintenance, the corresponding valve of the connecting pipe is opened, allowing water to flow from one pipe segment through a single pipe for transmission. This results in five specific maintenance conditions: 01#, 02#, 03#, 04#, 05#.

Due to the complexity of the actual engineering pipeline segment, parameters related to the project need to be set in the model. The specific parameter settings are shown in (**Table 1**), Parameters include four aspects: the material and properties of the simulated pipeline, the nature of the simulated liquid, the elevation of the upstream and downstream reservoirs, and the head supplied by the pumping station, which are defined in the mathematical model in order to accurately simulate the actual long-distance water transfer project. The water hammer wave speed in this pipeline segment is calculated as 818.82 m/s based on the pipe material, pipe length, burial method, and liquid properties.

**Table 1:** Engineering parameters.

Component	Parameters			
Pipeline	Material	Connected pipe length/Pipe length	diameter	Pipe wall thickness
	Pre-stressed Concrete Cylinder Pipe	10m /29.88km	3.0m	150mm
	Manning’s coefficient	Young’s modulus	Hazen-Williams coefficient	Poisson’s ratio
	0.013	2×10^7^ N/m^2^	110	15%
The simulated liquid	Type	Temperature	Kinematic viscosity	Bulk modulus of elasticity
	Water	4°C	1.566e^-0.006^ m^2^/s	2188.128 Pa
Reservoir	Upstream reservoir water level	Downstream reservoir water level		
	39.5m	45.5m		
Pumping station	Head			
	31.0m			

Based on the research objective of studying water hammer protection in asymmetric water transmission with pressurized dual-pipe segments, simulations need to be conducted for the cases of damage in each of the five segments. Additionally, in the 05# condition, where the water volume for one pipe segment is transported, without reaching the water transmission flow of the pipe segment, a 06# condition needs to be set for comparison. Valve closure times for each pipe segment are set to four different scenarios based on the length of the pipeline segment: T = 600 s, 300 s, 30 s, and 15 s. A total of 24 simulated scenarios. The specific condition settings are shown in (**Table 2**), Includes damaged pipe segments as well as the mode of operation and flow rate of the pipe segment setting.

**Table 2:** Operating conditions.

Project number	Operational status	Operation	Discharge
01#	Pipe 1 is damaged	Close the valve	17m^3^/s
02#	Pipe 2 is damaged	Close the valve	17m^3^/s
03#	Pipe 3 is damaged	Close the valve	17m^3^/s
04#	Pipe 4 is damaged	Close the valve	17m^3^/s
05#	Pipe 5 is damaged	Close the valve	8.5m^3^/s
06#	Pipe 5 is damaged	Close the valve	17m^3^/s

## 4 Model calculation

### 4.1 Normal water transmission in the pipeline

**Fig 8:**
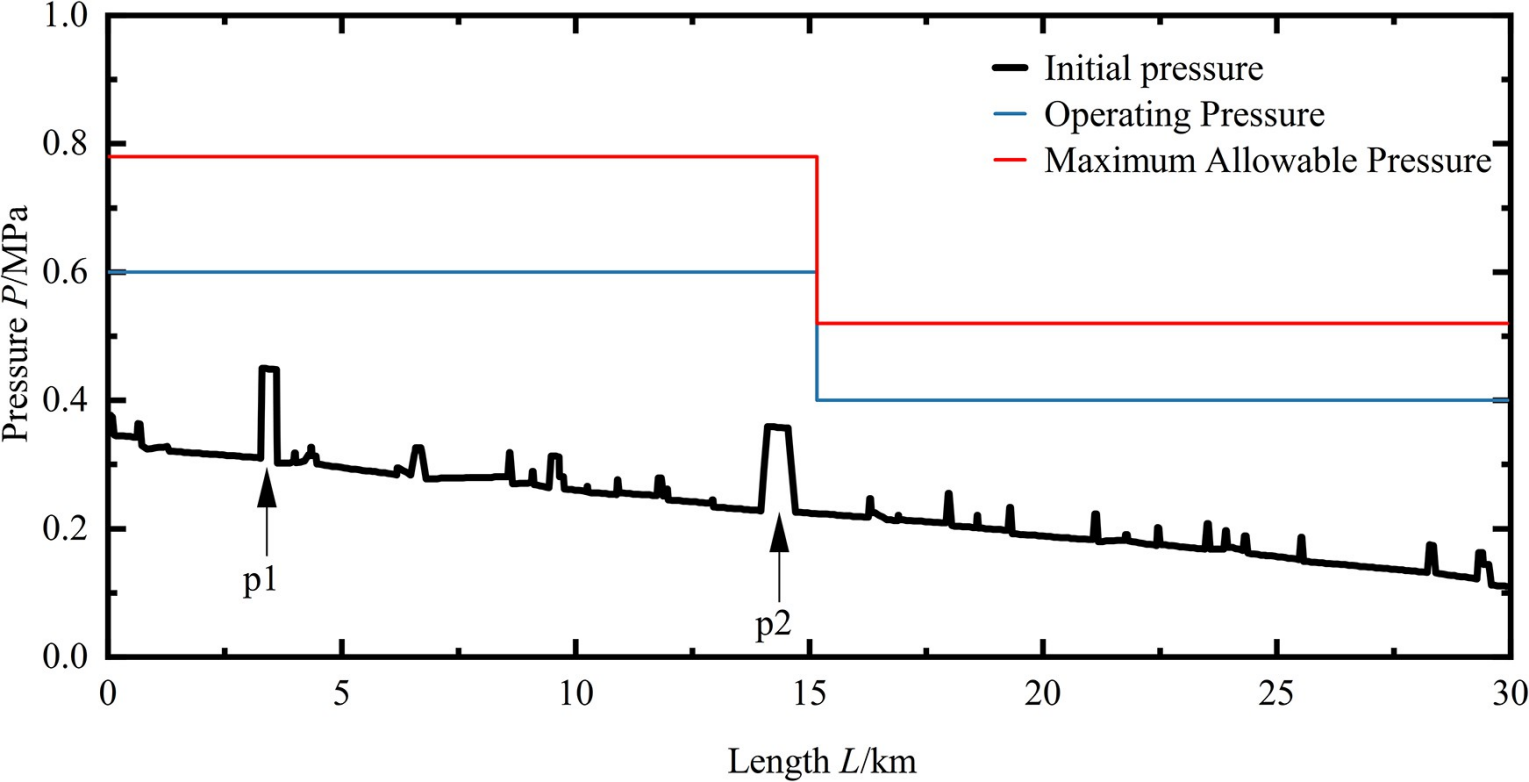
Pressure distribution along the pipeline under normal operating conditions.

When the pipeline is in a normal water transmission state, the pressure distribution inside the pipe is illustrated in **(Fig 8)**. There are two prominent peaks (P1, P2) in the maximum pressure of the pipeline. The sharp change in pressure at these points is due to the pipeline encountering a river. To avoid crossing the river directly, the pipeline needs to lower its elevation below the riverbed elevation in the vertical direction to navigate around the river. The reduction in the elevation of the pipe bottom results in an increase in the potential energy of the fluid within the pipeline. With the potential energy remaining constant, this leads to an increase in pressure energy. The maximum pressure in the pipeline occurs at P1 with a value of 0.448 MPa. Comparing this with the working pressure along the line (*P*_*D1*_ = 0.6 MPa), it is evident that the pipeline is within the working pressure range under normal operating conditions. According to the domestic outdoor water supply design standard GB 50013–2018, it is stipulated that the maximum pressure should not exceed 1.3 to 1.5 times the working pressure. Due to the slight difference in material between the front and back end of the pipeline during the construction of the pipeline, their ability to withstand the pressure of water is also different. Therefore, under abnormal operating conditions, the maximum pressure at the front part of the pipeline should be less than 0.78 MPa, set as *P*_*maxf*_ = 0.78 MPa, and the maximum pressure at the rear part of the pipeline should be less than 0.52 MPa, set as *P*_*maxb*_ = 0.52 MPa. The maximum pressure value that each node on the pipeline can withstand is denoted as *Pmax*. The maximum pressure value that each node on the pipeline can withstand is denoted as *Pmax*. This data is used to determine whether the pipeline is in a safe state under abnormal operating conditions. Additionally, the minimum pressure value generated within the pipeline can also reflect the situation of the pipeline under abnormal operating conditions. If the minimum pressure value is negative, there is negative pressure inside the pipeline, causing it to collapse inward. The generation of negative pressure can pose significant risks to the pipeline. Therefore, when studying water hammer protection for the pipeline’s valve closure, it is crucial to focus on the negative pressure generated in the pipeline under this specific condition and evaluate the effectiveness of water hammer protection measures in eliminating negative pressure.

### 4.2 Calculation without protective measures

Although this study simulated different protective measures for all six scenarios, scenarios 02# and 04# correspond to damage in the second and fourth sections of the pipeline, respectively. Their water hammer effects fall between the other scenarios and lack representativeness. Therefore, scenarios 01#, 02#, 03#, and 04# are selected for analysis. The analysis focuses on the maximum and minimum pressure values at nodes within all pipeline sections after water hammer occurs during valve closure. In this study, the maximum and minimum pressures refer to the maximum and minimum pressure values at nodes in the pipeline during the simulation period. The curve connecting the maximum pressure values for all nodes is referred to as the maximum pressure envelope curve, while the curve connecting the minimum pressure values is called the minimum pressure envelope curve.

### 4.3 Maximum pressure

**Fig 9:**
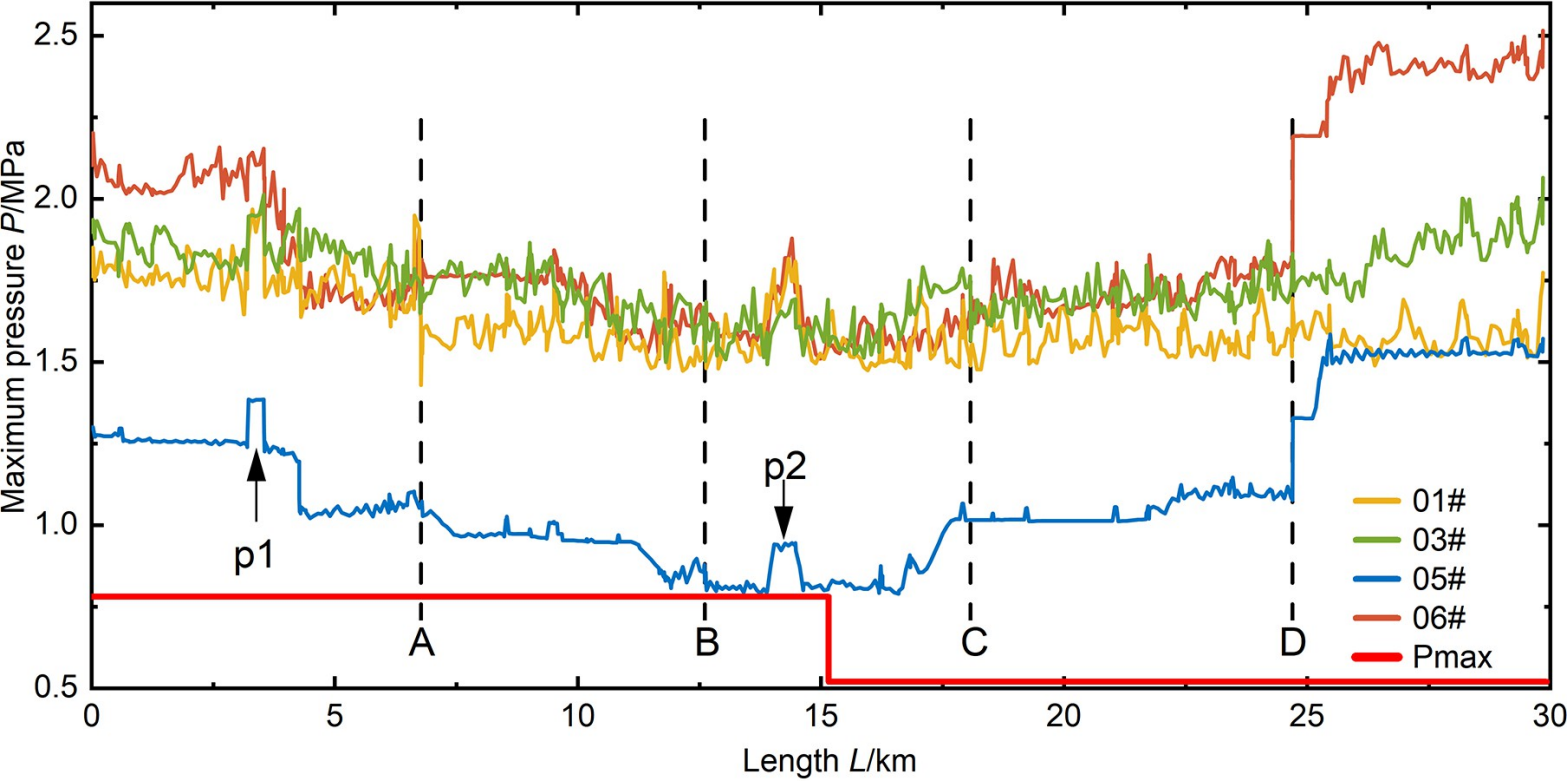
Pressure distribution along the pipeline for a valve closure time of 15 seconds.

**Fig 10:**
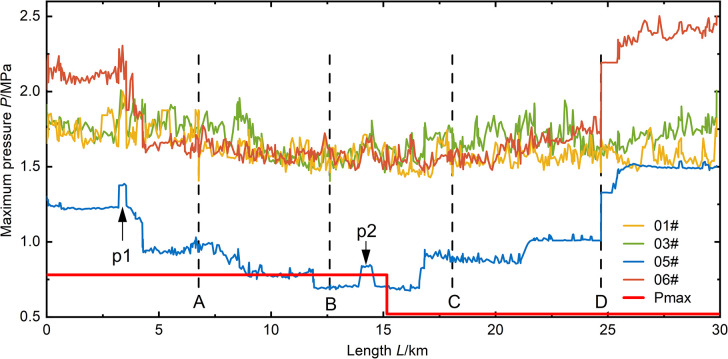
Pressure distribution along the pipeline for a valve closure time of 30 seconds.

**Fig 11:**
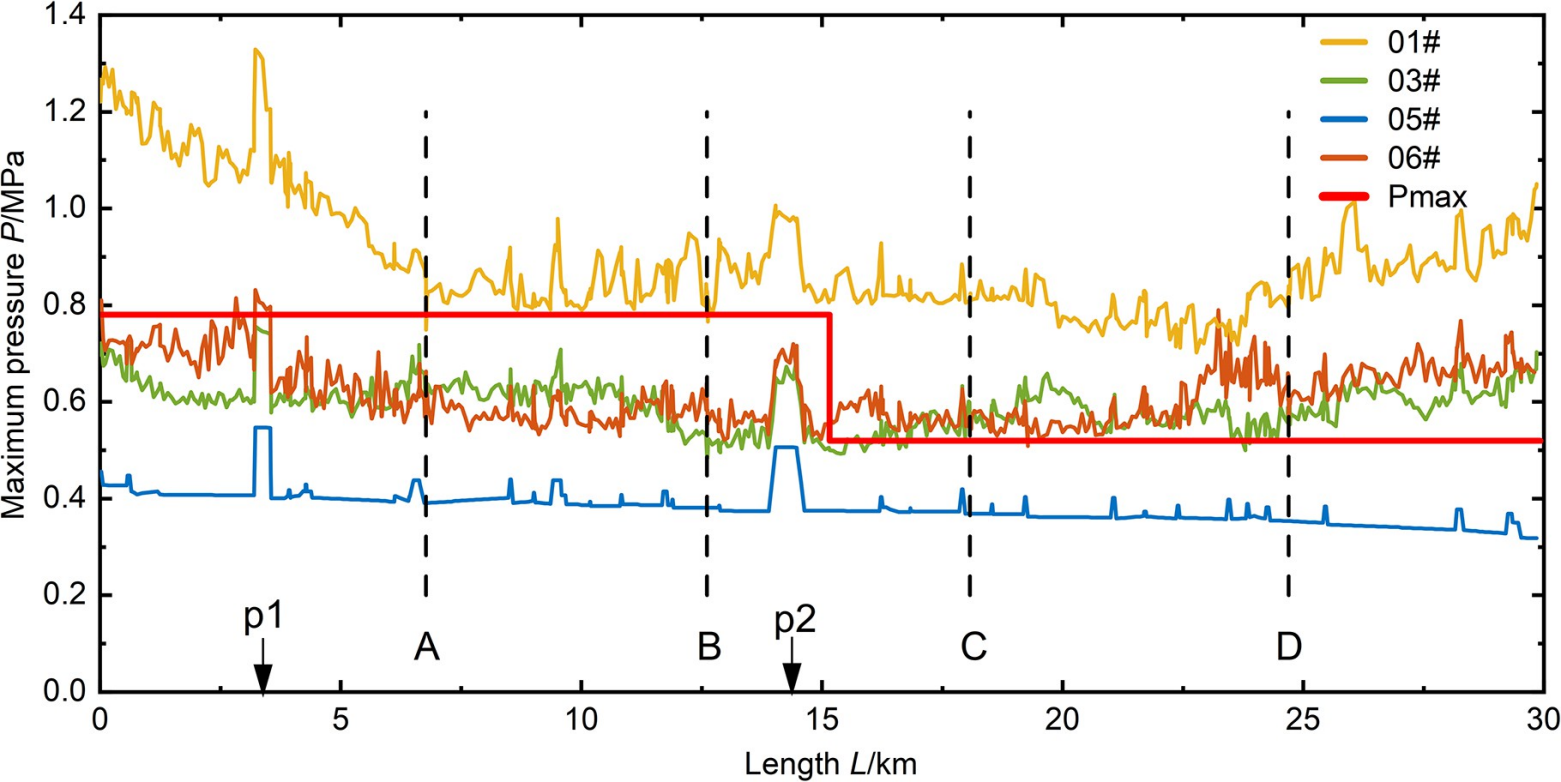
Pressure distribution along the pipeline for a valve closure time of 300 seconds.

**Fig 12:**
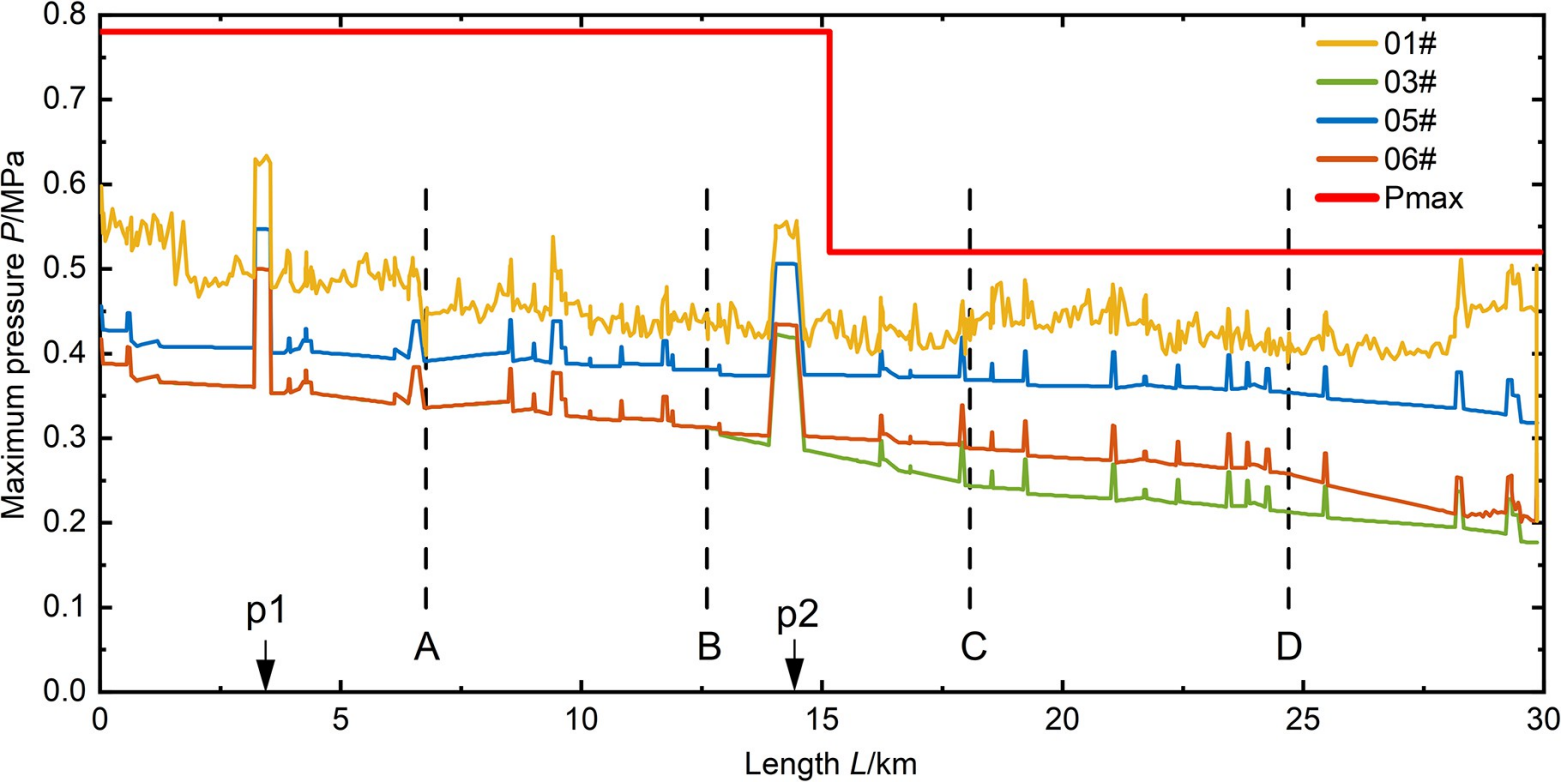
Pressure distribution along the pipeline for a valve closure time of 600 seconds.

As shown in **(Figs 9–12)**, it can be observed that in the case of a relatively short valve closure time, the maximum pressure in the pipeline due to water hammer decreases as the flow decreases. In the analysis of the 05# and 06# conditions at *T* = 15 s and 30 s valve closure times, when the water flow in the pipe is reduced to half of the normal flow, the maximum pressure in the pipeline is reduced by 40%. When the valve closure time is longer, the maximum pressure in the pipe drops to the steady-state initial pressure of the pipeline and cannot be further reduced. At this point, due to the higher flow rate in the 06# condition compared to the 05# condition, the initial pressure of the steady state is also higher, resulting in the pipeline pressure of 06# being higher than that of 05# in the case of a valve closure time of 600 seconds.

According to (**Figs 9–12)**, it can be observed that the position of opening the connecting pipe has a significant impact on the maximum pressure generated in the latter half of the overall pipeline after the section is damaged. For example, in the 05# and 06# conditions, where the pipeline is set to transport water when R5 (the fifth section of the right pipeline) is damaged, as shown in **(Fig 7)**, it is necessary to open connecting pipe D. It can be clearly seen that in both conditions of generating valve closure water hammer, the pressure in the downstream pipeline of the connecting pipe significantly increases. Comparing the water transport conditions in all scenarios, under the same flow rate of water transport, when the valve closure time is below 30s, the 06# condition is obviously the most unfavorable. When the valve closure time is long, reaching 300s, the 01# condition becomes the most unfavorable. If the valve closure time continues to increase, the maximum pressure values for all scenarios will approach the steady-state initial pressure, and at this point, the impact of the water hammer generated by all scenarios on the pipeline is relatively small.

In scenarios with a relatively small valve closure time, such as when the pipeline’s maximum pressure occurs at *T* = 15s, the maximum pressure values generated by the sections in all four scenarios exceed the pipeline’s maximum allowable pressure *Pmax*. When the valve closure time is *T* = 30s, except for some sections in the 05# scenario that are below *Pmax*, valve closure operations in other scenarios result in unfavorable pressures for pipeline operation. Comparing the valve closures at 15s and 30s, it can be seen that the two valve closure times have a relatively small impact on all scenarios, and the increase in time is not significant. This leads to minimal reduction in the maximum pressure, indicating that if there is a need to reduce the effect of valve closure water hammer, it is necessary to increase the valve closure time. When the valve closure time is *T* = 300s, except for the 05# scenario where the maximum pressure along the section is at the initial pressure, the transient impact of valve closure water hammer on the section is slow due to the low water flow rate in the 05# section. The maximum pressure values along the sections in other scenarios are all less than *Pmax*. When the valve closure time is *T* = 600s, the maximum pressure values generated in all scenarios are below *Pmax*, reducing the risk of pipe bursting during section operation.

**Fig 13:**
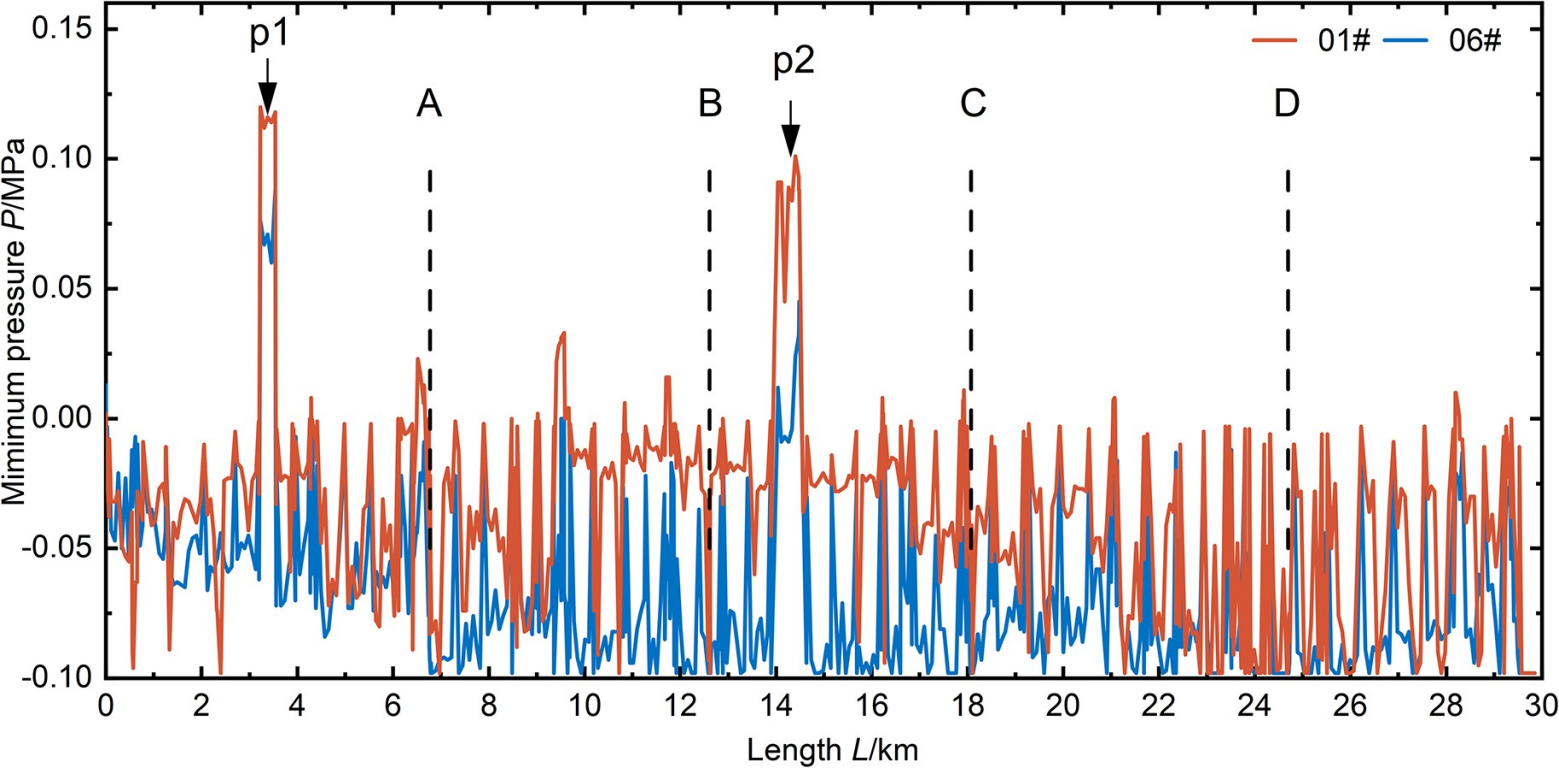
Minimum pressure distribution in the pipeline for a valve closure time of 600 seconds.

Since the sections can operate safely when the valve closure time is *T* = 600s, the minimum pressure values along the section during valve closure operations are shown in **(Fig 13).** For the 01# and 06# scenarios, except for points P1 and P2 where the sections pass underneath, other points along the sections experience minimum pressures below zero. In these areas, the probability of pipe collapse increases, posing a risk to the pipeline. Therefore, it is necessary to consider applying water hammer protection measures to the sections in the 01# and 06# scenarios to reduce the pressure on the sections.

### 4.4 Minimum pressure

The minimum pressure values under different operating conditions exhibit variations. To visually evaluate the change in the minimum pressure within the pipeline segment with respect to the closing time under different conditions, the number of nodes with negative values in the pipeline segment is counted and analyzed, as shown in (**Table 3**). In the established model, when the pressure at a node within the pipeline segment is less than -98 kPa, it is considered that water vaporizes at that node, and thus, the data is presented as -98 kPa. Based on the aforementioned statistical data, the count of nodes with a minimum pressure of -98 kPa within the pipeline segment is presented in (**Table 4**).

**Table 3:** Number of nodes with negative minimum pressure values for damage in different pipe sections at different shut-off times (Total number of nodes: 893).

Shut-off time	1#	2#	3#	4#	5#	6#
15s	398	572	691	329	696	632
30s	545	690	757	750	785	741
300s	874	861	854	865	776	864
600s	789	859	872	872	796	873
1200s	829	844	864	867	779	868

**Table 4:** Number of nodes with minimum pressure value of -98kpa damaged in different pipe sections at different shut-off times (Total number of nodes: 893).

Shut-off time	1#	2#	3#	4#	5#	6#
15s	60	306	283	56	40	244
30s	129	342	433	341	75	343
300s	527	433	284	390	331	302
600s	54	32	123	97	33	115
1200s	14	10	75	42	32	100

Based on the data from Tables 3 and 4, it can be observed that under different operating conditions, the number of nodes with negative pressure increases as the valve closure time extends. For instance, as shown in Table 3, when the valve closure time in condition 1# is 15 seconds, only 398 nodes within the pipeline exhibit negative pressure. However, when the valve closure time is extended to 300 seconds, the number of nodes with negative pressure increases to 874, with 98% of the pipeline nodes experiencing negative pressure. When the valve closure time is further extended to 1200 seconds, the number of nodes with negative pressure decreases slightly to 829. Despite this slight decrease, compared to the shorter closure times of 15 and 30 seconds, the number of nodes with negative pressure still shows a 100% increase.

Furthermore, as indicated in Table 4, when the valve closure time exceeds 300 seconds, the number of nodes with a pressure lower than -98 kPa decreases. For example, in condition 1#, when the valve closure time is 300 seconds, the number of nodes with a pressure below -98 kPa is 527. However, when the valve closure time is increased to 1200 seconds, this number drops to just 14 nodes.

Analyzing this alongside Fig 3, it becomes clear that when the valve closure time exceeds 300 seconds, the number of nodes with negative pressure increases, while the number of nodes with pressure lower than -98 kPa decreases. This suggests that as the valve closure time surpasses 300 seconds, the distribution of negative pressure within the pipeline becomes more uniform, reducing the occurrence of extreme pressure values and consequently lowering the risk of pipeline failure.

In previous simulations, it was observed that compared to placing a pressure relief well at the front or in the middle of the pipeline, positioning it at the end yielded the most effective pressure regulation results for the pipeline. Therefore, considering this finding, a decision was made to install a pressure relief well at the end of the pipeline in this project. A simplified design for the pressure relief well was chosen for implementation in the pipeline pressure regulation. The parameters for the pressure relief well were determined based on a review of past literature and practical engineering considerations, We decided to install a pressure regulating well with a height of 70m at the end of the pipeline at an elevation of 46.1m, and set the diameter of the pressure regulating well at 5m and the diameter of the connection hole at 2m. as outlined in (**Table 5**).

**Table 5:** Surge tank parameters.

Installation Location	Elevation of Foundation	Top Elevation	Pipeline Diameter	Surge Tank Diameter		Connection Aperture	
Pipeline Termination	46.1m	116.1m	3m		5m		2m

## 5 Results and discussion

To seek solutions for reducing the pipeline’s behavior during valve closure conditions, the primary objective is to minimize the maximum pressure generated within the pipeline. Additionally, efforts should be made to minimize the occurrence of column separation and water hammer effects within the pipeline during valve closure.

**Fig 14:**
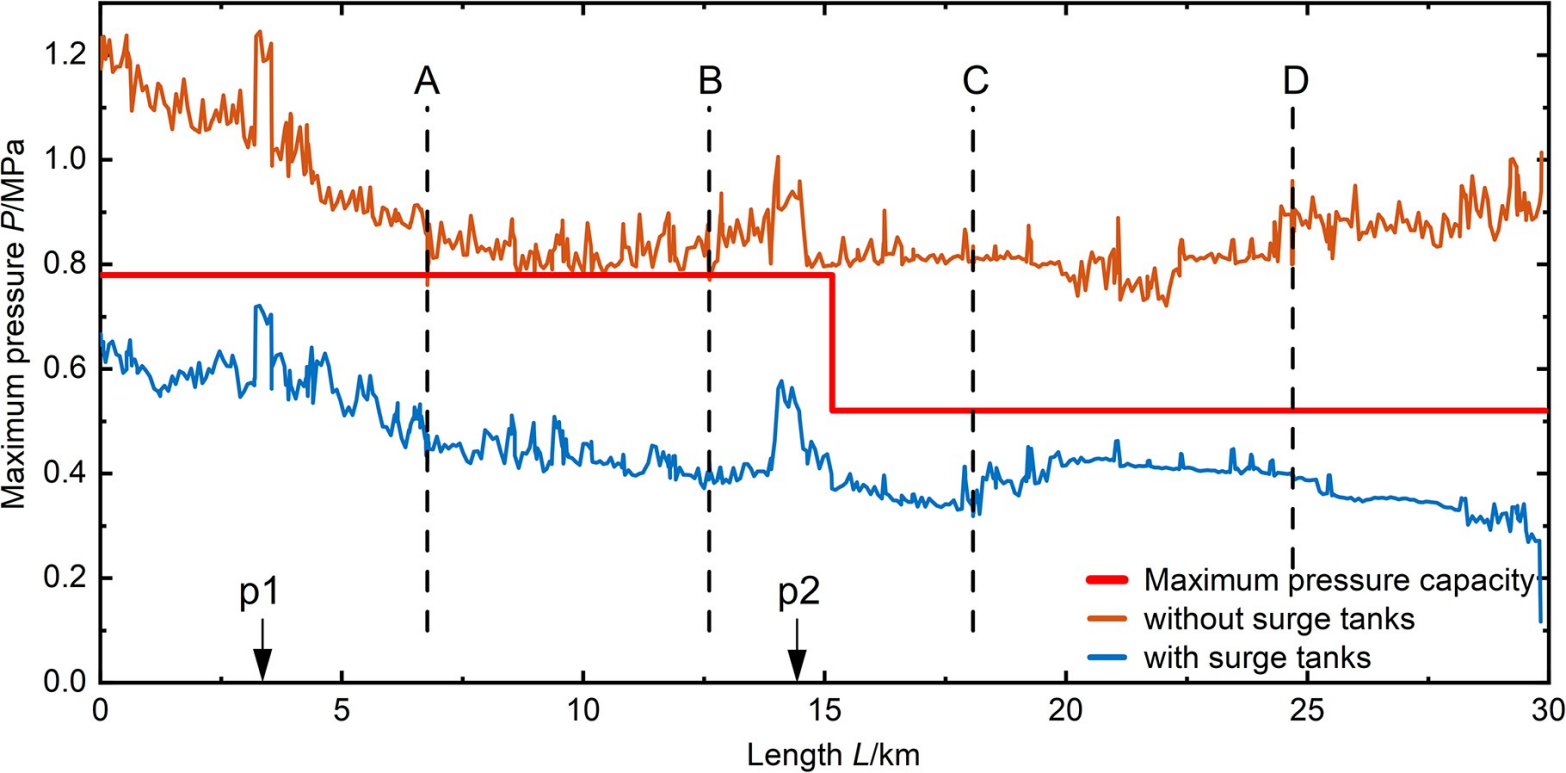
01# maximum pressure distribution in pipeline during valve closure (300s).

**Fig 15:**
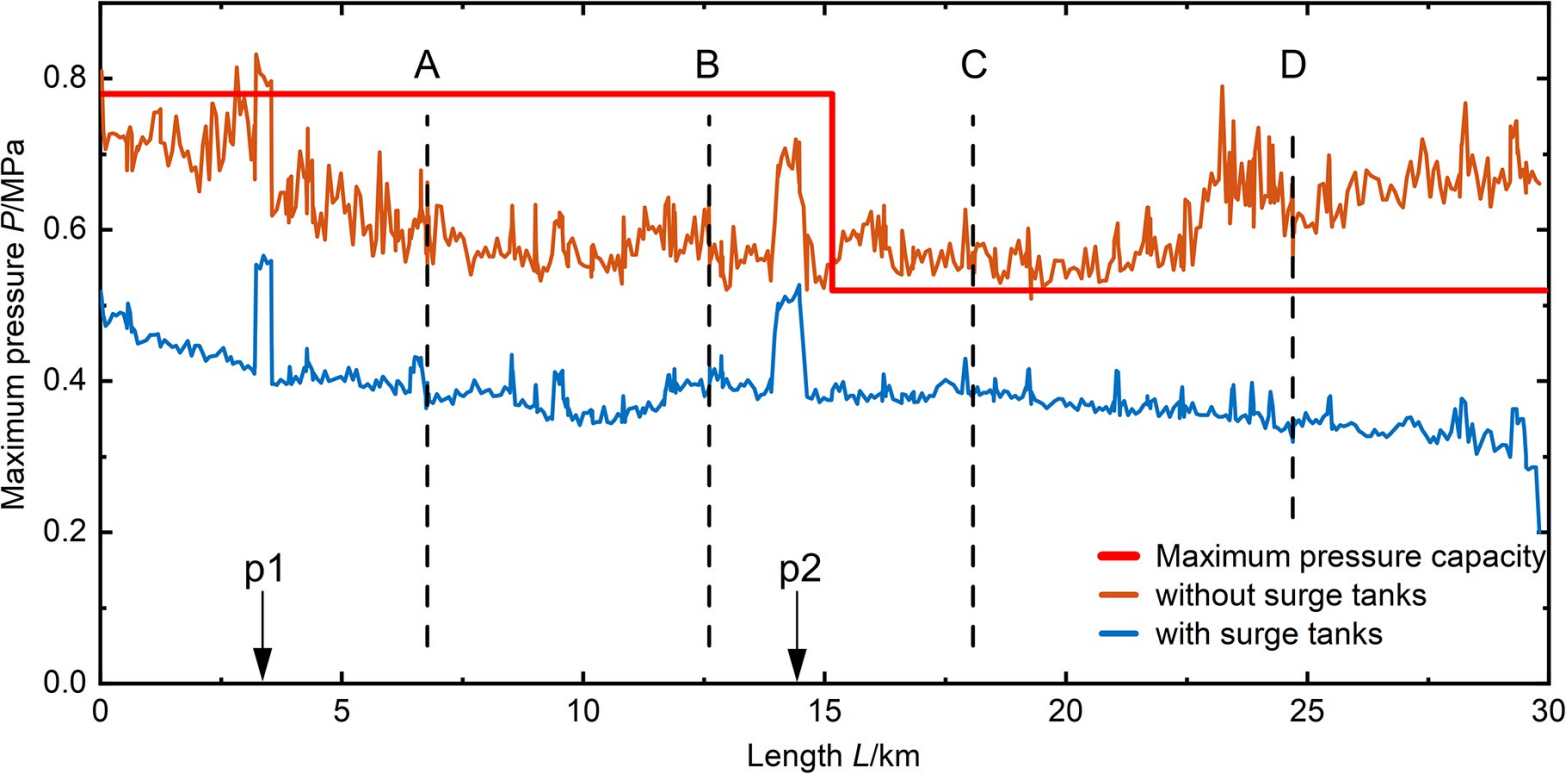
06# maximum pressure distribution in pipeline during valve closure (300s).

In the previous analysis, it is evident that increasing the closing time of the valve in various scenarios can reduce the maximum pressure values during transient changes in water flow within the pipeline. Furthermore, at a valve closing time of 600 seconds, the pipeline pressure reaches a minimum, and the number of nodes with minimum pressure values, either negative or below -98 kPa, is minimized within the pipeline. However, in practical engineering, a valve closing time of 600 seconds is quite long. To increase the efficiency of valve closing and provide protection for the pipeline segment, the results after installing a surge tank at the pipeline’s end based on a 300-second valve closing time are shown in **(Fig 14)**. It is evident that, after closing the surge tank at the end of the pipeline, in the 06# scenario, the maximum pressure within the pipeline segment decreases after valve closing, reducing from 832 kPa to 566 Kpa. In the 01# scenario, after the pipeline experiences valve closure water hammer, the maximum pressure within the pipeline segment also decreases, falling below the working pressure of the pipeline. Due to the fact that the 06# and 01# scenarios represent the most unfavorable conditions for maximum pressure, the decrease in pressure under these conditions results in a reduction in the maximum pressure for other scenarios, as shown in **(Fig 15).** This indicates an overall decreasing trend in the maximum pressure of pipeline segments, demonstrating an enhanced protective effect after installing the surge tank.

After installing the surge tank, according to (**Table 6**), it is observed that the 01# and 06# scenarios experience a reduction in the number of nodes where the pipeline segment’s minimum pressure becomes negative or falls below -98kPa. Therefore, the installation of the surge tank weakens the impact of valve closure water hammer at the end of the pipeline segment for these scenarios.

**Table 6:** The number of negative pressure nodes in the pipeline before and after installing the surge tank. (Total number of nodes: 893).

Operating condition		06#		01#	
	≤ 0 kPa		≤ -98 kPa	≤ 0 kPa	≤ -98 kPa
Without surge tank	853		300	871	526
With	839		176	836	231

## 6 Conclusions

1. In the scenario of valve closure for this dual-pipeline segment, an increase in flow leads to a decrease in pressure under steady-state conditions. However, after the occurrence of valve closure water hammer, the pressure inside the pipeline segment increases. Moreover, under the same valve closure time, damage to the end segment of the pipeline is most unfavorable for the occurrence of valve closure water hammer. Even in the pipeline segment with air valves installed, negative pressure still exists inside the pipeline.
2. After installing the surge tank at the end of the pipeline segment, the overall pressure in the pipeline segment decreases. The number of nodes with negative pressure in the pipeline segment also decreases, and the number of nodes below -98kPa is also reduced. Therefore, by increasing the valve closure time and adding a surge tank under this specific condition, the harm to the pipeline segment after the occurrence of valve closure water hammer is reduced.
3. The protective measures for the water hammer at the end of the double-pipe water delivery system studied in this research involve closing the valve for more than 300 seconds and installing a surge tank. This can reduce the pipeline’s overpressure and certain negative pressure, decreasing the likelihood of liquefied air in the pipeline and safeguarding the pipeline segment. The results can provide some references for future research on protection and the design of measures for dual-pipe water conveyance systems.

## Supporting information

S1 File (ZIP)
